# High resolution mapping of restoration of fertility (*Rf*) by combining large population and high density genetic map in pigeonpea [*Cajanus cajan* (L.) Millsp]

**DOI:** 10.1186/s12864-020-06859-6

**Published:** 2020-07-03

**Authors:** Rachit K. Saxena, Johiruddin Molla, Pooja Yadav, Rajeev K. Varshney

**Affiliations:** 1 International Crops Research Institute for the Semi-Arid Tropics (ICRISAT), Hyderabad, 502324 India; 2 Ghatal Rabindra Satabarsiki Mahavidyalay, Ghatal, Paschim Medinipur 721212 India

**Keywords:** Pigeonpea, Hybrids, Restoration of fertility, Genetic map, QTL, Candidate gene

## Abstract

**Background:**

Restoration of fertility (*Rf*) is an important trait for pigeonpea hybrid breeding. Few coarse quantitative trait locus (QTL) studies conducted in the past identified QTLs with large confidence intervals on the genetic map and could not provide any information on possible genes responsible for *Rf* in pigeonpea. Therefore, a larger population comprising of 369 F_2_s derived from ICPA 2039 × ICPL 87119 was genotyped with high density Axiom *Cajanus* SNP Array with 56 K single nucleotide polymorphism (SNPs) for high resolution mapping of *Rf*.

**Results:**

A genetic map with 4867 markers was developed and a total of four QTLs for *Rf* were identified. While one major effect QTL (*qRf*8.1) was co-localized with the QTL identified in two previous studies and its size was refined from 1.2 Mb to 0.41 Mb. Further analysis of *qRf*8.1 QTL with genome sequence provided 20 genes including two genes namely flowering locus protein T and 2-oxoglutarate/Fe (II)-dependent dioxygenases (2-ODDs) superfamily protein with known function in the restoration of fertility.

**Conclusion:**

The *qRf*8.1 QTL and the potential candidate genes present in this QTL will be valuable for genomics-assisted breeding and identification of causal genes/nucleotides for the restoration of fertility in the hybrid breeding program of pigeonpea.

## Background

Success of the three-line hybrid breeding system i.e. cytoplasmic genetic male sterility system (CGMS) in any crop species depends on the stability of male sterility and extent/frequency of restoration of fertility (*Rf*). It is also important to note that *Rf* is crucial in influencing the yield of hybrid plants. Some studies in past indicated that *Rf* is affected by both minor and major effect quantitative traits loci (QTLs) in pigeonpea [*Cajanus cajan* (L.) Millsp.] [1, 2]. High resolution mapping, fine mapping and subsequently cloning of these QTLs to identify the genes will be useful for pigeonpea improvement. The importance of high resolution mapping and fine mapping has been realized in a number of crop species for several disease resistance and agronomic traits [3–8]. Moreover, QTLs/ genes associated with *Rf* have been isolated in few crops and vegetable species including barley [9], maize [10] and Chinese cabbage [11].

In the case of pigeonpea, majority of QTL studies were based on relatively small population size and a few hundred molecular markers [1, 2]. As a result, QTLs identified in the previous studies were quite big with a confidence interval ranged from 7 cM to 23 cM [1, 2]. Such QTLs, though serve the purpose of genomics assisted breeding (GAB) if they explain higher phenotypic variation (PVE), can also bring linkage drag during their introgression in the elite varieties. Furthermore, high resolution mapping of such big QTLs can help identification of candidate genes with causal polymorphism with a trait to develop diagnostic markers and precise transfer of gene/small genomic region for the trait in the elite varieties. In general, the size of QTL and the extent of PVE of the QTLs mainly depend on: (i) size and the trait variation in the mapping population, and (ii) marker density on the genetic map. Limited or less number of individuals in a given segregating/mapping population may lead to biases in QTL mapping studies. It may affect the mapping, PVE and accuracy of QTL. To overcome the limitation of population size in QTL mapping, a number of approaches have been proposed in several studies [12–15]. One of these approaches is to include more individuals in a given population for high resolution mapping after a coarse QTL study [13].

Another key factor in determining the QTL size is the number of molecular markers mapped on to the genetic map used for QTL study. High-density genetic map can localize recombination events more precisely and would help in reducing the QTL size or identification of new QTLs that were not identified with sparse genetic map. Recent advances in next generation sequencing (NGS) and high-throughput genotyping have facilitated development of high density genetic maps in different crop species including pigeonpea [2]. Such high density genetic maps can be used for identification of QTLs in smaller window providing higher resolution of QTLs.

Considering the importance of above mentioned two points in QTL detection i.e. population size and high density genetic maps, high resolution mapping studies have been conducted in several crops like Brassica [5, 7]), cotton [5], chickpea [16] and rice [3, 4]. For instance, in the case of chickpea, high-density bin map was used to fine map the “*QTL-hotspot*” region. In fact, “*QTL-hotspot*” region was split into two sub regions and candidate genes for drought tolerance were identified in this study [16]. Similarly, in maize, high density genotyping and large recombinant inbred population were used for the identification of QTLs for plant architecture related traits [17]. Therefore, in the present study, we have performed high-throughput SNP genotyping using the Axiom *Cajanus* SNP Array with 56 K SNPs [18] on extended population of 369 F_2_s derived from ICPA 2039 × ICPL 87119 to construct a high-density genetic map and undertake high resolution mapping of QTL region associated with *Rf* in pigeonpea.

## Result

### High density genetic map

**Fig. 1:**
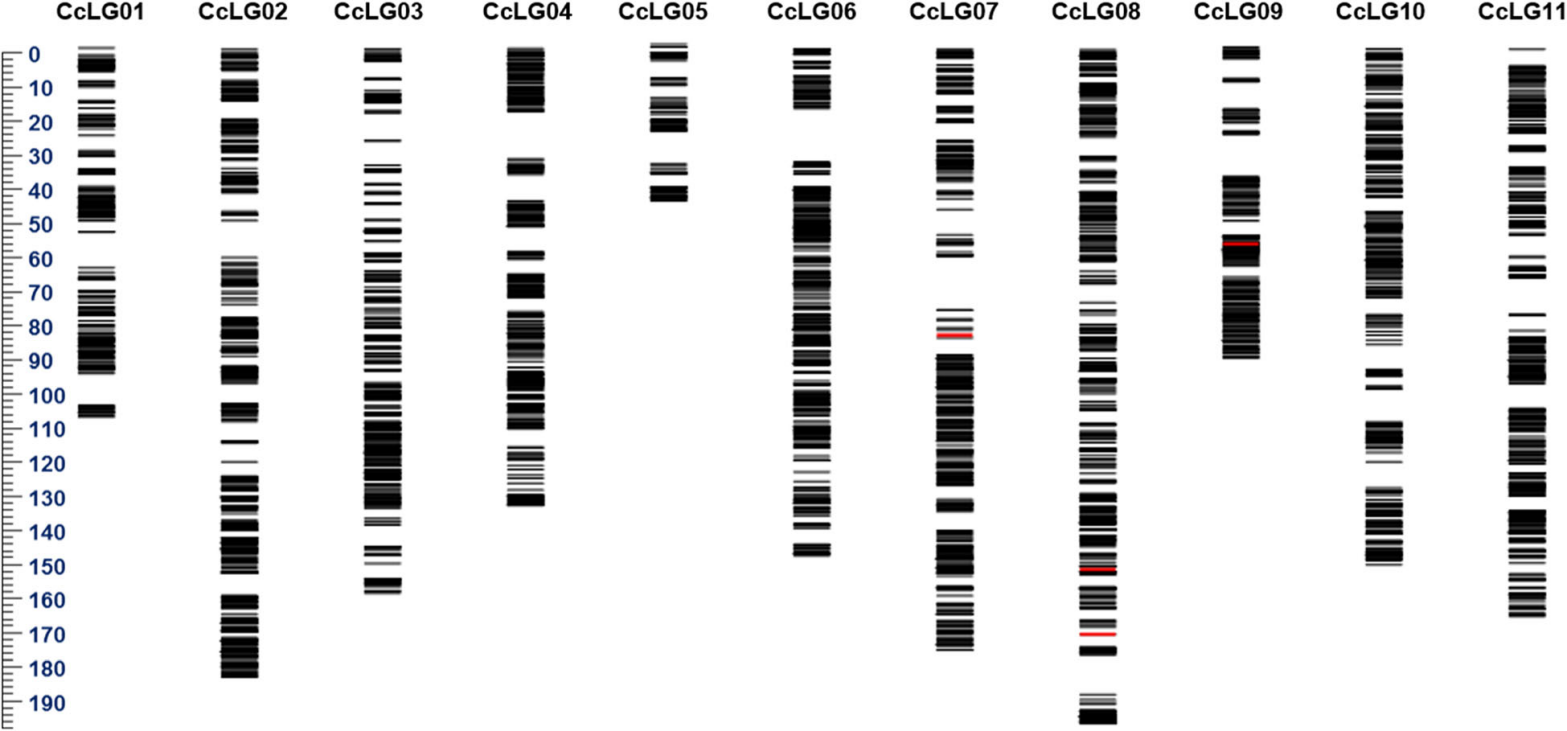
High density genetic map with 4867 SNPs distributed on 11 CcLGs for an enlarged F_2_ (369 individuals) population segregating for restoration of fertility in pigeonpea

High-quality genotyping data were generated on 369 F_2_s along with parental lines using 56 K Axiom *Cajanus* SNP Array. These 56 K SNPs were having their pre-defined positions on the pigeonpea genome and more details on this SNP Array could be seen in Saxena et al. [18]. In summary, high- quality data were obtained for a total of 56,512 SNPs. SNP genotyping data were further subjected to different filtering parameters such as missing data points, heterozygous and monomorphic SNPs. Finally, a set of 12,079 SNPs showed polymorphisms between the parents of the mapping population. The identified polymorphic SNPs were evaluated for expected Mendelian segregation ratios through Chi-square analyses. The SNPs with distorted segregation ratio were removed. As a consequence, 7711 SNPs were used for the construction of genetic map. The final genetic map was comprised of 4867 SNPs distributed on 11 CcLGs (Fig. 1 and Table 1). The genetic map encompassed 1580.68 cM, with 11 CcLGs ranging from 50 cM (CcLG05) to 197.1 cM (CcLG08). The lowest and highest number of SNPs were mapped on CcLG05 (115 SNPs) and CcLG02 (719 SNPs), respectively. But the percentage of SNPs mapped from the total polymorphic SNPs was highest on CcLG04 (86.3%) and lowest on CcLG11 (41.73%). Whereas, lowest marker density was found on CcLG01 and CcLG05 (2.3 markers/cM), and highest marker density was on CcLG02 (3.91 markers/cM).

**Table 1:** Distribution of SNP markers on the genetic map for ICPA 2039 × ICPL 87119 (F_2_) population

CcLG	SNPs (polymorphic)	SNPs (1:2:1) (P-value of < 10^− 4^)	SNPs mapped	SNPs mapped (%)	Mapping length (cM)	Number of markers per cM
CcLG01	1106	466	256	54.93	111.3	2.3
CcLG02	1871	1231	719	58.4	184.06	3.91
CcLG03	1219	802	400	49.87	160.59	2.49
CcLG04	708	482	416	86.3	136.16	3.06
CcLG05	199	157	115	73.24	50	2.3
CcLG06	1157	731	546	74.69	150.18	3.64
CcLG07	1000	677	513	75.77	176.64	2.9
CcLG08	1056	734	631	85.96	197.1	3.2
CcLG09	617	426	281	65.96	94.87	2.96
CcLG10	1154	716	452	63.12	152.54	2.96
CcLG11	1992	1289	538	41.73	167.24	3.22
Total	12,079	7711	4867	729.97	1580.68	3.07
Average	1098.09	701	442.45	66.36	143.69	2.99

### Fine mapping of *Rf*

**Fig. 2:**
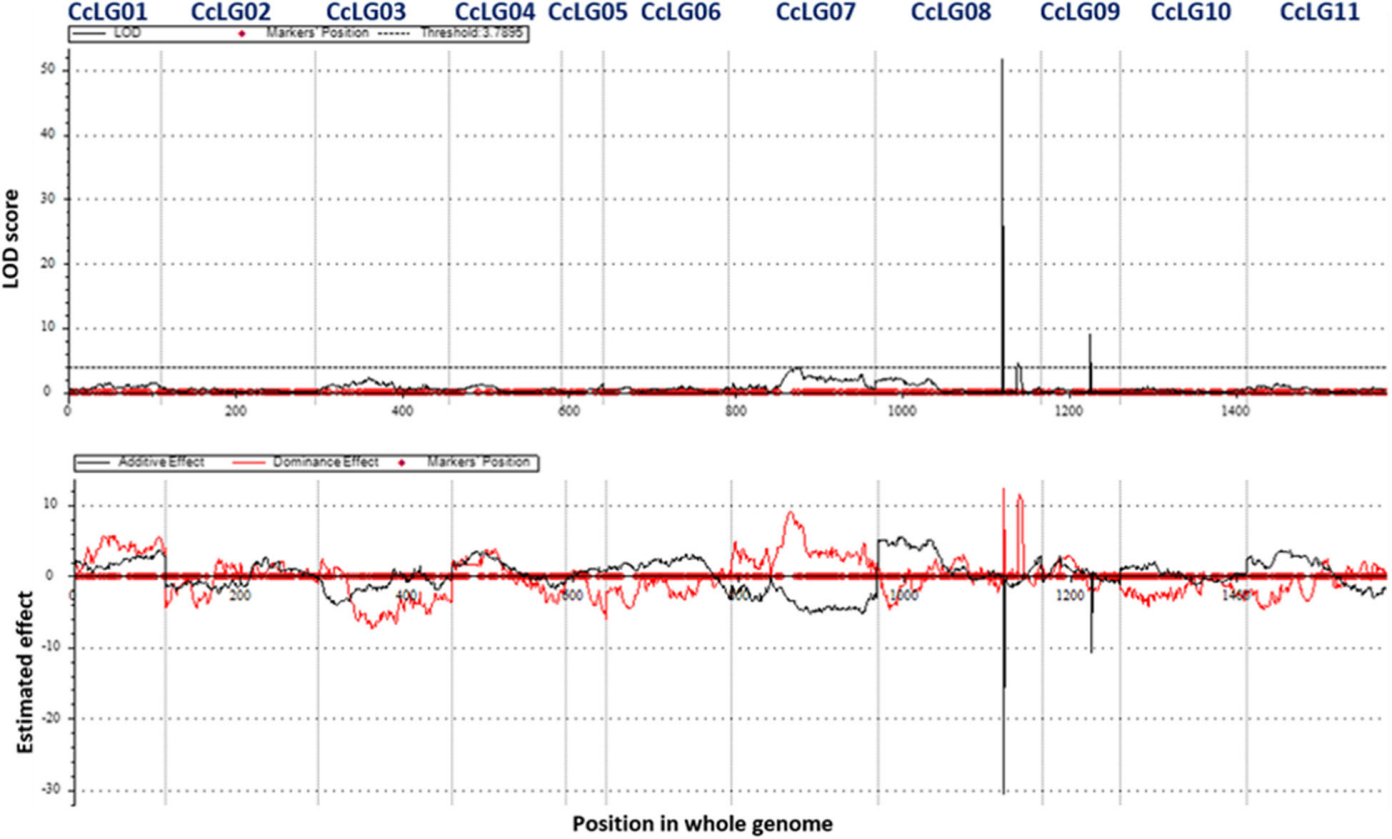
A major QTL (*qRf8.1*) flanked by Affx-123,357,076 to Affx-123,360,361 on CcLG08 with PVE 45.06% at LOD value 51.58

High density genotyping data generated in the present study together with previously generated phenotyping data [2] for *Rf* were used for the QTL analysis. Though we have used all 415 F_2_s for DNA isolation but high quality genotyping data could be generated on 369 F_2_s and parental lines. ICIM analysis provided a total of four QTLs for *Rf* distributed on 3 CcLGs (Table 2). Identified QTLs were designated as *qRf7*, *qRf8.1*, *qRf8.2*, and *qRf9*. The *qRf7* flanked by Affx-123,311,044 - Affx-123,323,924 on CcLG07, *qRf*8.1 flanked by Affx-123,357,076 - Affx-123,360,361, *qRf*8.2 flanked by Affx-123,318,646- Affx-123,334,846 on CcLG08 and *qRf*9 flanked by Affx-123,344,569- Affx-123,309,157 on CcLG09. The identified QTLs with PVE equal to or higher than 10% were considered as major QTL and those showing less than < 10% considered as minor QTL. The PVE of the QTLs was in the range from 2.34% (*qRf*7) to 45.06% (*qRf*8.1). In terms of localization of QTLs on CcLGs, the CcLG08 contained one major QTL namely *qRf*8.1 (PVE 45.06%) (Fig. 2) and one minor QTL, *qRf*8.2 (PVE 2.79%). While the remaining two minor QTLs were located one each on CcLG07 (*qRf*7 with PVE 2.34%) and on CcLG09 (*qRf*9 with PVE 5.78%) (Table 2).

**Table 2:** Summary of QTL mapping for *Rf* derived from F_2_ (ICPA 2039 × ICPL 87119)

QTL	CcLG	Position (cM)	Marker interval		QTL size (Mb)	PVE%	LOD value	Additive effect
			Genetic map	Genome				
*qRf*7	CcLG07	84	Affx-123,311,044 - Affx-123,323,924	3,491,625–5,770,779	2.2	2.34	3.84	−4.71
***qRf*8.1**	CcLG08	152	Affx-123,357,076 - Affx-123,360,361	7,706,211–7,295,478	0.41	45.06	51.58	−30.63
*qRf*8.2	CcLG08	171	Affx-123,318,646 - Affx-123,334,846	4,209,765–3,895,976	0.31	2.79	4.45	−0.88
*qRf*9	CcLG09	60	Affx-123,344,569 - Affx-123,309,157	8,976,251–8,933,062	0.04	5.78	8.99	−10.83

### High resolution mapping and candidate genes for *Rf*

In the past, only two studies have been conducted to locate the QTL regions associated with *Rf* in pigeonpea using different marker systems and mapping populations [1, 2]. Therefore, to understand the advances achieved through high resolution mapping, results from this study were compared with the results obtained in previous studies. The first study was based on low density genetic maps derived from 78 to 140 simple sequence repeat (SSR) markers and four segregating populations with 188 F_2_ individuals each. This study identified a total of six major-effect QTLs across three populations. The PVE of identified QTLs was in the range of 14.85 to 24.17% [1]. In the second study, we used 306 SNPs based genetic map and 186 F_2_s for *Rf* mapping [2]. This study provided one major QTL flanked by S8_7,664,779 to S8_6,474,381 SNPs in 70.5 cM – 90.8 cM confidence intervals on CcLG08 with PVE 28.5% and LOD 8.7 [2]. The results obtained through SSRs and SNPs from these two studies were also compared by mapping them on to the pigeonpea genome in this study. It was found that most of the major QTLs detected by Bohra et al. [1] were co-located with major QTL identified on CcLG08 by Saxena et al. [2]. The mapped positions of above mentioned QTLs were corresponding to 2.0 Mb [1] and 1.2 Mb [2] of pigeonpea genome.

**Fig. 3:**
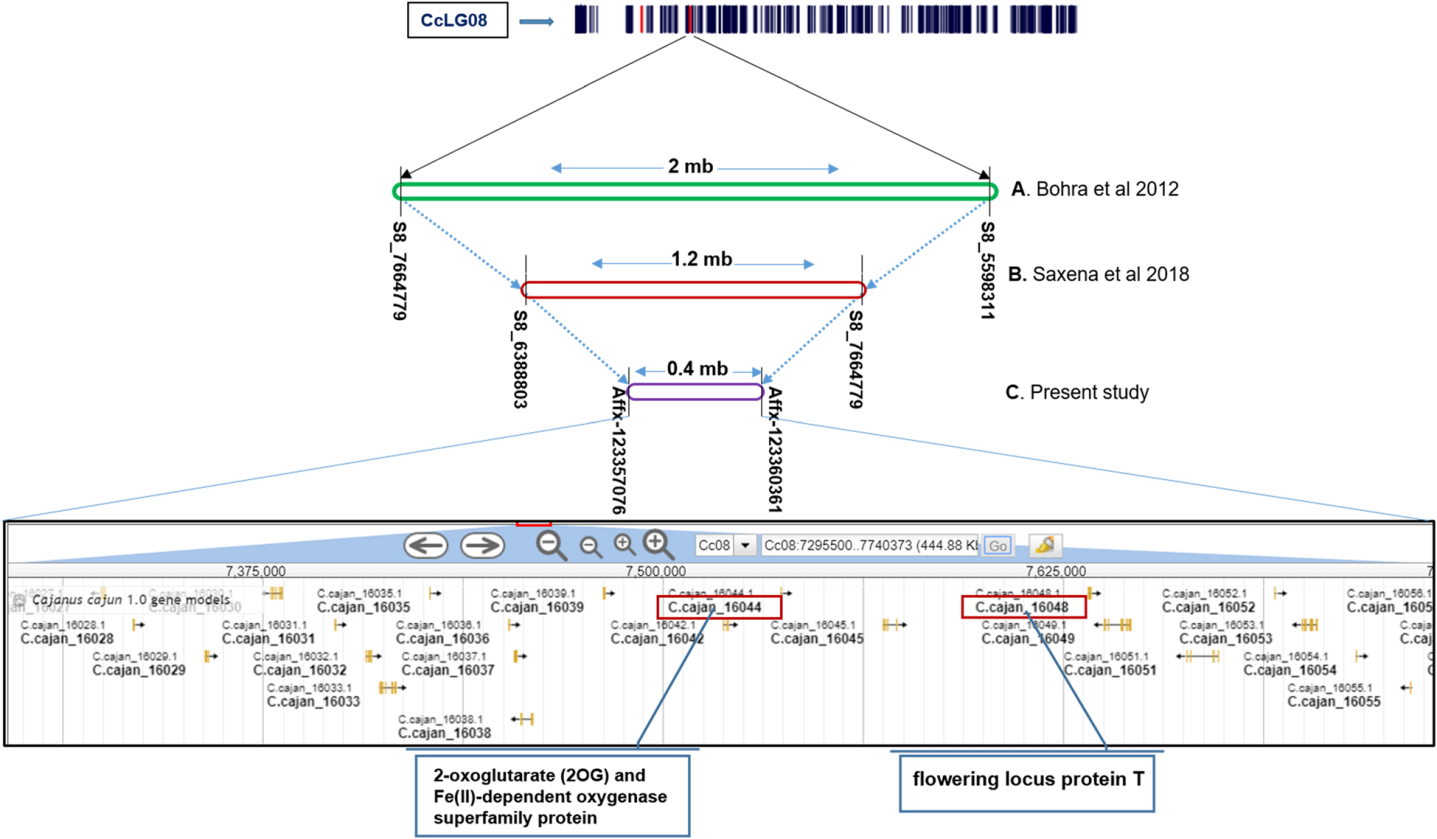
Comparisons of the *Rf* associated QTLs identified in Bohra et al. [1] and Saxena et al. [2] with the present study and candidate genes detected

In the present study using 56 K *Cajanus* SNP Array and 369 F_2_s, one major QTL (*qRf*8.1) flanked by Affx-123,357,076 to Affx-123,360,361 on CcLG08 was identified. Phenotypic variance explained by *qRf*8.1 was highest (45.06%) across all the QTLs detected so far in different studies with LOD value 51.58. Moreover, QTLs positions on the reference genome across three studies were compared. It is important to note that major QTLs detected in past two studies either collocated or overlapping with the *qRf*8.1. The present study could reduce the possible target genomic region for *Rf* from 2 Mb [1] and 1.2 Mb [2] to just 0.41 Mb (Fig. 3, Table 3). In order to remove the software biasness while comparing the QTL sizes in present and previous SNPs based studies [2], earlier generated data with GBS based SNPs were also used for QTL mapping using ICIM with same parameters mentioned in the methods section. The QTL region identified with the ICIM was almost similar in size (1.27 Mb) as of identified through QTL cartographer in our previous study [2]. Further, target genomic region in *qRf*8.1 was also searched for the presence of candidate genes. A total of 20 candidate genes were found in *qRf*8.1(Supplementary Table 1). Out of 20 candidate genes, two candidate genes namely flowering locus protein T and 2-oxoglutarate/Fe (II)-dependent dioxygenases (2-ODDs) superfamily protein have shown their role in restoration of fertility in different crop species [19, 20].

**Table 3:** Mapping and QTL comparison of present study with previous studies

Population size	Mapped markers	Average inter marker distance			LOD	PVE %	References
		Genetic map (cM)	Genome position	QTL size (Mb)			
188	78–140 SSRs	7.3–3.1	5,598,311 to 7,664,779	2	8.9	13.98–24.17	Bohra et al. 2012 [1]
186	306 SNPs	3.2	6,474,381 to 7,664,779	1.2	8.7	28.5	Saxena et al. 2018 [2]
369	4867 SNPs	0.3	7,295,478 to 7,706,211	0.41	51.58	45.06	Present study

## Discussion

In a number of crop species including pigeonpea large amount of genomic resources have become available [18, 21–25]. These genomic resources are being used in understanding and validating the basic biological, evolutionary concepts to downstream application in crop improvement and seed systems. In order to deploy genomic resources for crop improvement, marker-trait associations need to be established and subsequently causal mutations or genes need to be identified. However, a large amount of genomic and genetic resources coupled with good strategies and commitment is required to go from association to causality in the genome for a given trait. Therefore, as a quick fix, marker- trait associations are established and used in GAB. Nevertheless, for precise selection of line for a trait in crop improvement programs, it will be good to identify causal mutation or QTLs in a minimum confidence interval. This will also help to avoid the issue of linkage drag during the transfer of QTLs in elite varieties.

High resolution mapping is an approach which can be used for shortening the large genomic segments identified in coarse QTL mapping [16]. Extending the population size and high-density genetic maps could improve the QTL mapping resolution [26, 27]. In the present study 369 F_2_s and their genotyping with 56 K SNPs array as well as phenotyping data were used. This population has more than double individuals as compared to previously used population in coarse *Rf* QTL studies in pigeonpea [2]. Moreover, the genetic map developed in this study has around 16 to 62 fold higher markers density as compared to previous genetic maps used for *Rf* QTL studies in pigeonpea [1, 2]. Furthermore, the major QTL “*qRf*8.1” has contributed almost half of the phenotypic variation 45.06% with LOD 51.6. Enhanced confidence level in terms of LOD and PVE values in the present study may be due to our high resolution mapping approach where large population has been used. Similar observations were made in maize, murine where LOD value considerably increased with the size of mapping population [28, 29].

All the *Rf* associated QTLs detected in this study including minor and major had significant additive effects for the trait. As expected, the alleles for increasing traits value were from ICPL 87119 for *qRf7*, *qRf8.1*, *qRf8.2 and qRf9* (Table 2). These mono parental additive effects suggest that the selection of ICPL 87119 as restorer parent was very appropriate and this line has a great potential to be used as one of the best restorers in pigeonpea hybrid breeding program. The high resolution mapping of *Rf* in the present study has also provided 20 potential candidate genes within major QTL-region (Supplementary Table 1). Among them two genes- flowering locus protein T (FT) and 2-oxoglutarate/Fe (II)-dependent dioxygenases (2-ODDs) superfamily protein play an important role in fertility [19, 20]. The FT belongs to the FT-like family, which includes at least six close FT-like genes (FT1 to FT6) in wheat [30–32]. Recently it has been reported that FT1 and FT2 play an important role in spike development and fertility [20]. The other protein 2-oxoglutarate/Fe (II)-dependent dioxygenases (2-ODDs) superfamily protein plays an important role on auxin concentration. This protein converts IAA into inactive oxIAA that in turn controls auxin concentration and homeostasis which is essential for reproductive development, including anther dehiscence, pollen fertility in rice and barley.

## Conclusion

In summary, the present study has used high resolution mapping by combining a large population and high density markers. Besides the increased resolution of genetic map and QTL region, several genes with known functions have been identified. With the enhanced confidence and multiple validations across different mapping populations used in past and present studies, flanking markers of “*qRf*8.1” can be used in GAB. Furthermore, potential candidate genes in this QTL region will be helpful in identification, cloning and functional validation of causal mutation or gene/s responsible for *Rf* in pigeonpea.

## Methods

### Mapping population and DNA isolation

For mapping *Rf,* one F_2_ mapping population was developed from CMS line ICPA 2039 and its known fertility restorer ICPL 87119 [2]. The seeds of crossing parental lines were obtained from ICRISAT pigeonpea improvement program. However only a subset of this population i.e. 186 F_2_s was used in coarse QTL mapping for *Rf* [2]. In the present study, however, all 415 F_2_s from the above mentioned population were used.

Young leaves from individual plants and parental lines were used to isolate genomic DNA using NucleoSpin plant extraction kit (MACHEREY-NAGEL, USA). The quality and quantity of DNA were checked on 0.8% agarose gel and NanoDrop™ 8000 Spectrophotometer (Thermo Scientific, US), respectively.

### Genotyping and construction of genetic map

DNAs from 415 F_2_s were used for high density genotyping through 56 K Axiom *Cajanus* SNP Array as described in Saxena et al. [18]. However, high quality genotyping data were used for only 369 F_2_s for construction of the genetic map. In this regard, called SNPs with respect to the two parents were first tested against the expected Mendelian segregation ratios (1:2:1). SNPs following expected segregation ratio at a *P*-value of < 10^− 4^ were retained and used for the construction of genetic map.

Genotyping data were assembled for all segregating markers on all F_2_ individuals and linkage analysis was performed using regression mapping algorithm by ICIM software v4.1 [33]. Map calculations were performed with default parameters and in addition of a new locus may influence the optimum map order; hence, a “Ripple” was used. The Kosambi mapping function was used to convert recombination fraction into map units [34]. The visualization of genetic map was done using the software ICIM software v4.1 and MapChart 2.32 [35].

### QTL analysis and candidate genes

QTL mapping for *Rf* was done by combining phenotyping data [2] together with SNP data generated in the present study. ICIM-ADD method with default parameters were used to detect QTL using ICIM software v4.1. The threshold of the LOD score for declaring the presence of a significant QTL was determined by the permutation test with 1000 repetitions at *P* < 0.05. The nomenclature of QTLs was given as described in Yadav et al. [36]. Where QTLs include “*q*” for quantitative trait followed by trait code (“*Rf*” for restoration of fertility) with *Cajanus cajan* linkage group (CcLG) number and chronological order of QTL for that trait on the CcLG separated by a dot. Further, candidate genes present within the major QTL region were retrieve from *Cajanus cajan* 1.0 gene model using JBrowse of legume information system (LIS). The function of these genes were predicted from gene search tool of LIS.

## Supplementary information

**Additional file 1: Table 1.** Potential candidate genes identified in major QTL region.

## Data Availability

All the data generated or analyzed during this study have been included in the present article as Tables and Figures.

## Abbreviations

CcLG: *Cajanus cajan* linkage group
cM: Centimorgan
DNA: Deoxyribonucleic acid
ICIM: Inclusive composite interval mapping
LOD: Logarithm of the odds
PVE: Phenotypic variance explained
QTL: Quantitative trait loci
SNP: Single nucleotide polymorphism
